# The transcriptional events and their relationship to physiological changes during poplar seed germination and post-germination

**DOI:** 10.1186/s12864-019-6180-5

**Published:** 2019-11-04

**Authors:** Chunpu Qu, Hancheng Zhao, Jinyuan Chen, Zhuang Zuo, Xue Sun, Jiahuan Huang, Chengjun Yang, Xiuli Zhang, Peng Zhang, Xiankui Quan, Zhiru Xu, Guanjun Liu

**Affiliations:** 1 State Key Laboratory of Tree Genetics and Breeding (Northeast Forestry University), School of Forestry, Northeast Forestry University, Harbin, 150040 People’s Republic of China; 2 School of Forestry, Northeast Forestry University, Harbin, 150040 People’s Republic of China; 3 College of Life Science, Northeast Forestry University, Harbin, 150040 People’s Republic of China

**Keywords:** Poplar, Seed germination and post-germination, RNA-Seq, Metabolite changes, Transcriptional timetable, Physiological index

## Abstract

**Background:**

Seed germination, the foundation of plant propagation, involves a series of changes at the molecular level. Poplar is a model woody plant, but the molecular events occurring during seed germination in this species are unclear.

**Results:**

In this study, we investigated changes in gene transcriptional levels during different germination periods in poplar by high-throughput sequencing technology. Analysis of genes expressed at specific germination stages indicated that these genes are distributed in many metabolic pathways. Enrichment analysis of significantly differentially expressed genes based on hypergeometric testing revealed that multiple pathways, such as pathways related to glycolysis, lipid, amino acid, protein and ATP synthesis metabolism, changed significantly at the transcriptional level during seed germination. A comparison of Σ*Z* values uncovered a series of transcriptional changes in biological processes related to primary metabolism during poplar seed germination. Among these changes, genes related to CHO metabolism were the first to be activated, with subsequent expression of genes involved in lipid metabolism and then those associated with protein metabolism. The pattern of metabolomic and physiological index changes further verified the sequence of some biological events.

**Conclusions:**

Our study revealed molecular events occurring at the transcriptional level during seed germination and determined their order. These events were further verified by patterns of changes of metabolites and physiological indexes. Our findings lay a foundation for the elucidation of the molecular mechanisms responsible for poplar seed germination.

## Background

Seed germination, which comprises a series of ordered physiological and morphogenetic processes beginning with seed imbibition, is the starting point for higher plant growth and development [1–6]. Strictly speaking, seed germination extends from the time of soaking until the hypocotyl has completely punctured through the outer layer of the embryo. A more general definition of seed germination includes the seed post-germination period, that is, the point at which the hypocotyl has completely penetrated the outer embryo layer until the cotyledon has fully expanded [1, 6, 7]. The propagation of most flowering plants depends on seeds. Successful seed germination is thus the basis of plant propagation and extremely important for global ecological stability [6].

Seed germination begins with the imbibition of water by dried seeds. Seed germination is divided into three periods based on rates of change of seed fresh weights during this process: stage 1, rapid water absorption; stage 2, slow water absorption and stage 3, water absorption during plant growth. The post-germination period includes seed hypocotyl elongation and cotyledon development [1, 2, 7–9].

Seed germination involves a series of complex changes in gene transcription and translation and protein modification. The emergence of high-throughput sequencing technology has allowed insights into molecular-level changes that occur during such complex processes. In recent years, seed germination processes have been explored in species such as Arabidopsis [10–12], soybean [13] and rice [14], with only a few such studies focusing on woody plants. For example, Zhang et al. used proteomics methods to reveal that energy dependence, protein synthesis and degradation, cell defense and rescue-related pathways are significantly correlated with poplar seed vigor [15]. Dewan et al. studied the development of black poplar seeds in Europe and concluded that maternal temperature and seed germination efficiency are closely related [16]. The molecular characteristics of seeds of woody plants at different stages of germination, especially the woody model plant poplar, have not been studied in detail.

Poplar is the common name for members of the genus *Populus*. These deciduous trees have a number of attractive characteristics, including rapid growth, environmental adaptability, superior genetic malleability, amenability to vegetative propagation, and a clear genomic background [17]. Because poplar reproduces vegetatively quite readily, little research has been carried out on its sexual reproduction [18, 19]. From the perspective of species evolution and environmental adaptation, however, long-term asexual reproduction is extremely unfavorable. Knowledge of the molecular mechanisms underlying seed germination in poplar seeds is key to elucidation of the molecular events occurring during this process.

Our research group has previously done some research on the molecular mechanism of poplar seed germination. For example, WGCNA are used to find the most relevant gene modules in the two periods of slow water absorption and cotyledon development, and the related genes are compared and analyzed [20]. However, for the complicated process of poplar seed germination, there is no report yet what changes have occurred in the transcriptional level in different periods of germination, which brings us a lot of troubles in the subsequent study on the molecular mechanism of poplar seed germination.

In this study, we therefore used high-throughput sequencing to analyze the transcriptomes of poplar at six different seed germination stages. Bioinformatics and mathematical statistical approaches were applied to reveal the biological events occurring at these different stages, which were further verified by examining changes in metabolomics and physiological indexes. Our results lay a foundation for understanding the molecular mechanisms underlying poplar seed germination.

## Results

### Identification of genes specifically expressed at different germination stages

**Fig. 1:**
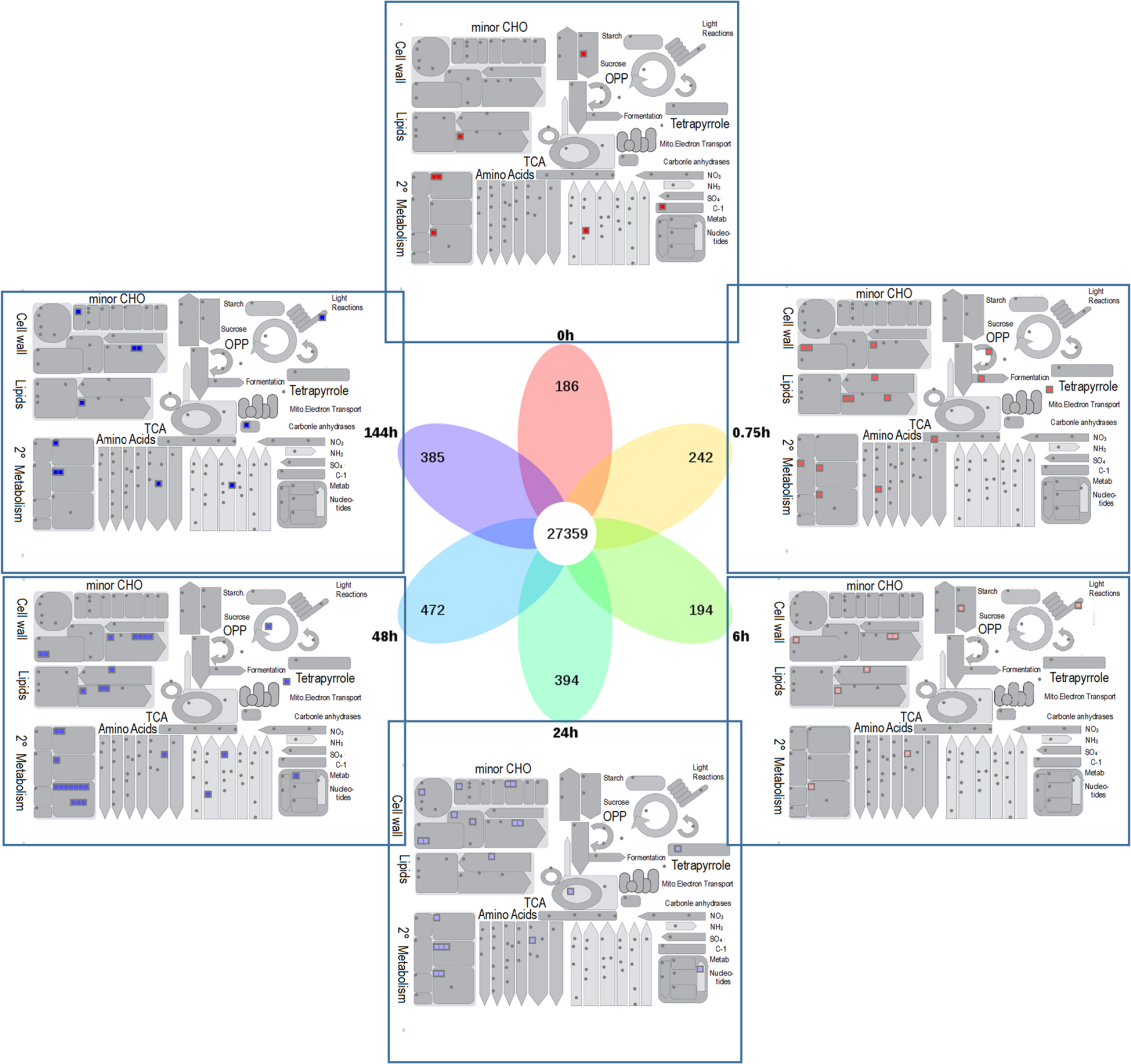
Mapman analysis of genes specifically expressed during different stages of poplar seed germination. Different color squares represent genes from different periods

A total of 27,359 differentially expressed genes were identified during poplar seed germination, which differ significantly from 0 h expression in at least one period. As shown in the Venn diagram in Fig. 1, 186, 242, 194, 394, 472 and 385 genes were specifically expressed at 0, 0.75, 6, 24, 48 and 144 h, respectively. Specifically expressed genes at each stage and their associated metabolic pathways are listed in Additional file 1: Table S1.

### Analysis of enriched Mapman categories during the germination process

**Fig. 2:**
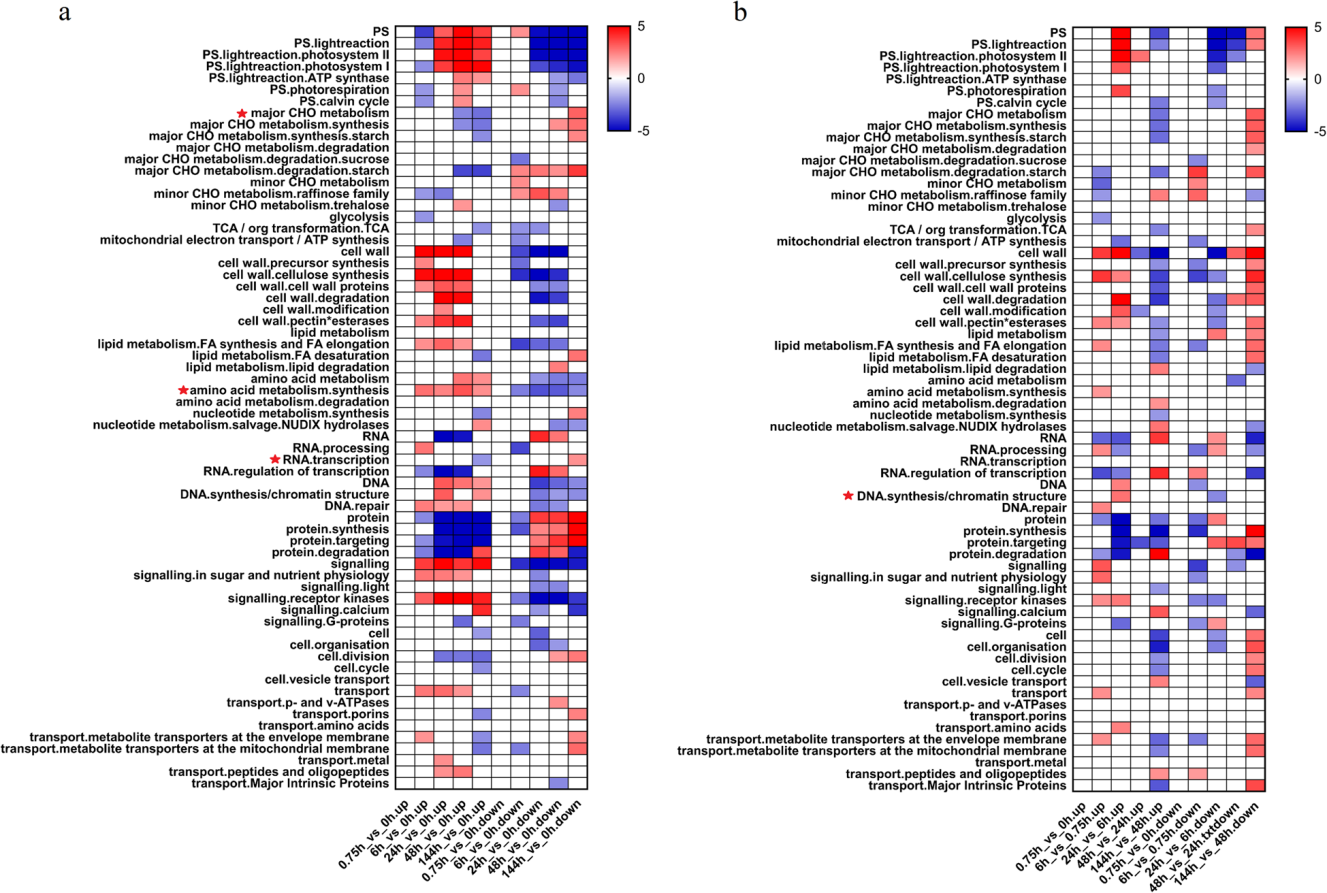
Enriched Mapman categories at each poplar seed germination stage. All significantly differentially expressed genes were categorized as up- or down-regulated and input into Mapman, and the degree of enrichment of each biological pathway was represented by a *Z*-value. Differentially expressed genes were compared in two ways: (**a**) using 0 h as the reference point and (**b**) using each previous time point in turn as the reference point. The scale of color represents the level of Z-values

To understand transcription-level changes in various biological pathways during poplar seed germination, all significantly differentially expressed genes were subjected to an enrichment analysis based on hypergeometric testing. The differentially expressed genes were compared in two main ways: 1) using 0 h as the reference point (fixed reference system) and 2) by selecting each previous adjacent time point in turn as the reference point (continuous comparison system). The results of the enrichment analysis are shown in Fig. 2, and all *Z*-value data are listed in Additional file 2: Table S2. When 0 h was used as the reference point, as shown in Fig. 2a, up-regulated genes in the major CHO metabolism pathway were significantly underrepresented at 48 and 144 h, while down-regulated genes in this pathway were significantly overrepresented at 144 h. Under the category of RNA transcription, up-regulated genes were significantly underrepresented and down-regulated genes were significantly overrepresented at 144 h. Up-regulated genes associated with amino acid synthesis were first significantly overrepresented at 6 h, which indicates that the amino acid synthesis pathway had undergone significant changes in activity at that time.

A heatmap of enriched Mapman categories based on the continuous comparison system is shown in Fig. 2b. The results of this analysis were basically consistent with those generated using a fixed reference point, but some of the trends were slightly different. According to the continuous comparison approach, for example, DNA synthesis-related up-regulated genes were only significantly overrepresented at 24 h, while down-regulated genes were underrepresented at 6 h. The differences in the number of enriched differentially expressed genes between adjacent and non-adjacent time periods indicate that the pattern of metabolic pathway changes fluctuated.

### The effect of the different comparison methods on the outcome of the enrichment analysis

**Fig. 3:**
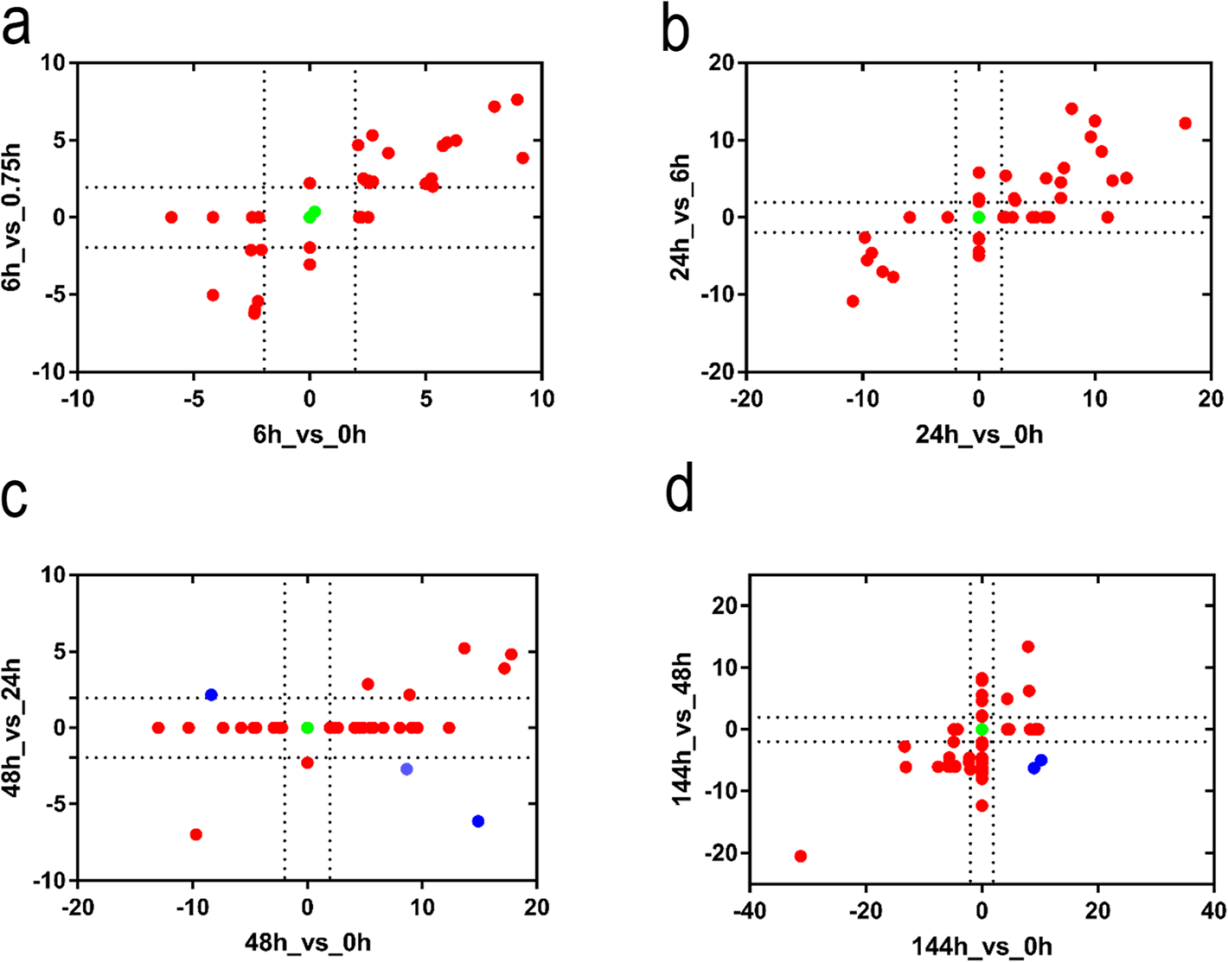
The effect of two differentially expressed gene comparison methods on the results of Mapman enrichment analysis. Data points are relative *Z*-values plotted on an x,y-coordinate system, where *x* corresponds to the *Z*-value obtained using 0 h as the reference point, and *y* corresponds to the *Z*-value calculated using each previous time point in turn as the reference. The vertical and horizontal dotted lines respectively denote *x*- and *y*-value significance thresholds (− 1.96 or 1.96). The color of each data point signifies the following: green, |*x*| and |*y*| ≤ 1.96 (not significant); red, |*x*| and/or |*y*| ≥ 1.96 (significant); blue, |*x*| and/or |*y*| ≥ 1.96 and the *Z*-value is in either the first or third quadrants. (**a**) (6h_vs_0h) vs (6h_vs_0.75h), (**b**) (24h_vs_0h) vs (24h_vs_6h), (**c**) (48h_vs_0h) vs (48h_vs_24h), (**d**) (144h_vs_0h) vs (144h_vs_48h)

A *Z*-value is a quantitative indicator of the number of standard deviations (σ = 1) of a variable from the mean of a standard normal distribution. When the number of up-regulated genes (the actual value) is larger than the number of background genes (the expected values), the *Z*-value is also larger; if the number of up-regulated genes is instead smaller, the *Z*-value is also smaller. The same is true for down-regulated genes. If the number of up-regulated genes in a given pathway is larger than the number of down-regulated ones, the pathway can be considered to be active; the converse is true for an inactive pathway. To assess whether specific pathways were active, we used relative *Z*-values (Σ*Z*), where Σ*Z* = *Z*_up-regulated genes -_ *Z*_down-regulated genes_. To determine how the different comparison methods (Fig. 2a and b) affected the enrichment analysis results, we generated plots in which the x-axis represented relative *Z*-values based on 0 h as the reference and the y-axis corresponded to relative *Z*-values using one of the previous time points as the reference. In other words, the relative *Z*-value from the two different comparison methods for each pathway corresponded to a unique point in the coordinate system, designated as *Z*(x, y)_relative value_. Most of the *Z*(x, y)_relative value_ points were in the first and third quadrants, which indicates that the two different comparison methods produced similar conclusions (Fig. 3a, b). In Fig. 3c and d, a few *Z*(x, y)_relative value_ points were in the second and fourth quadrants; these were primarily biological processes associated with cell wall degradation and photoreaction pathways (Additional file 3: Table S3). Overall, the results of the two comparison methods were not significantly different.

### Activity profiling of primary metabolic pathways at the transcriptional level

**Fig. 4:**
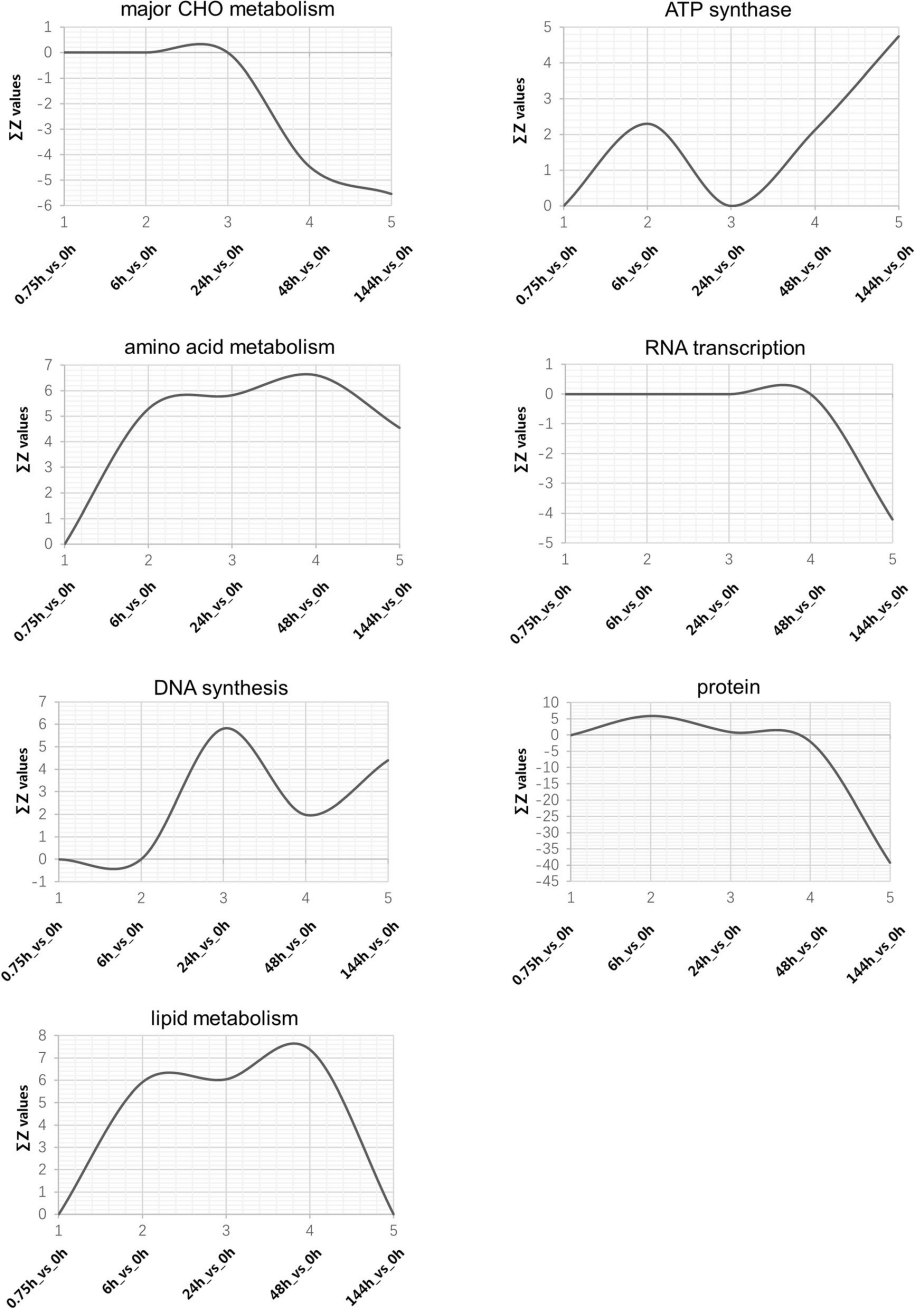
Changes in the activity of primary metabolism-related genes during poplar seed germination. The x-axis labels 1–5 are 0.75 h vs. 0 h, 6 h vs. 0 h, 24 h vs. 0 h, 48 h vs. 0 h and 144 h vs. 0 h, respectively, and the y-axis corresponds to Σ*Z* values

The activity levels of some primary metabolic pathways, including major CHO metabolism, ATP synthesis, amino acid metabolism, RNA transcription, DNA synthesis, protein, and lipid metabolism pathways, are shown in Fig. 4. As can be seen in the figure, CHO metabolism activity was highest at the earliest stage of poplar seed germination and then decreased after 24 h. The transcriptional activity of the ATP synthesis pathway fluctuated during seed germination. The first peak was at approximately 6 h, and the second peak was after 48 h. The transcriptional activity of the amino acid metabolism pathway exhibited an increasing trend during seed germination, with the highest activity at 48 h. The transcriptional activity of the protein pathway was always higher during seed germination and then disappeared after 48 h. Consistent with the transcriptional activity of the protein pathway, the RNA transcription pathway was highly active throughout seed germination until 48 h. DNA synthesis displayed high transcriptional activity at 24 h, and the activity of the lipid metabolism pathway was high from 6 to 48 h.

### Primary metabolism-related genes and their expression patterns

**Fig. 5:**
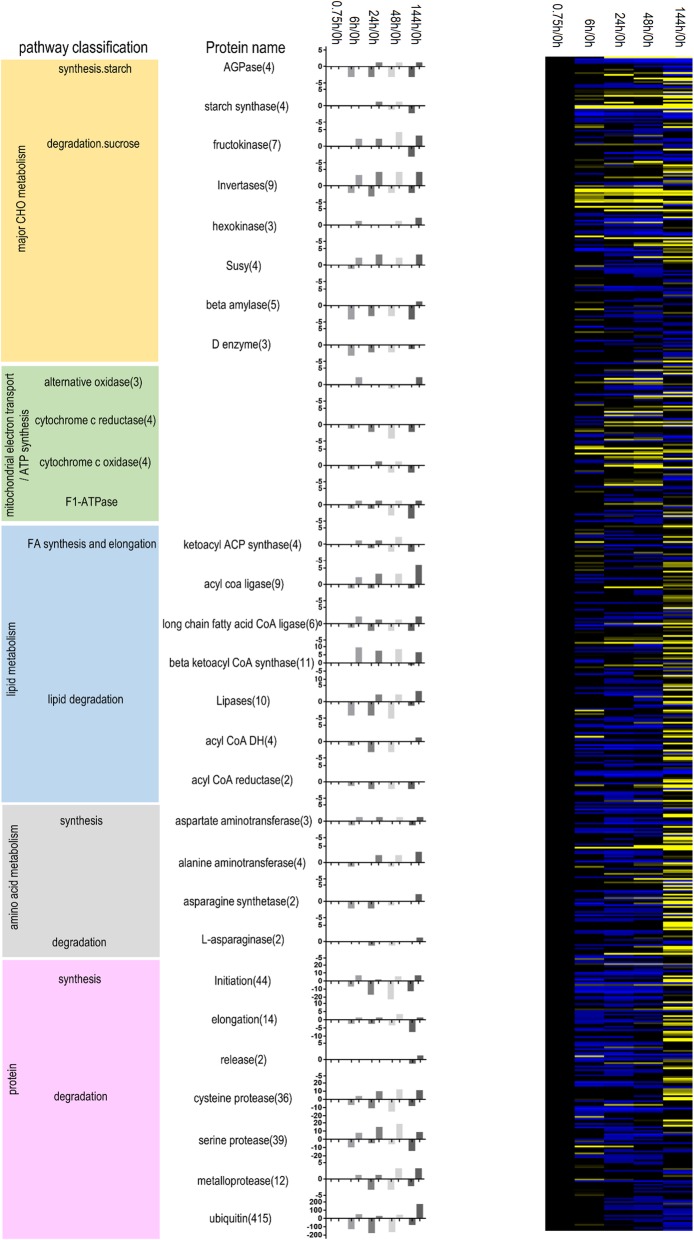
Primary metabolic pathway genes identified in this study. Pathway classifications, annotations, up- and down-regulated genes at different stages and an expression heat map are shown. Expressions: blue, minimum; yellow, maximum

Primary metabolic processes, such as major CHO metabolism, lipid metabolism, protein metabolism, amino acid metabolism and ATP synthesis metabolism, play an important role in seed germination. The genes and expression patterns of these pathways are listed in Fig. 5. Two enzymes involved in starch synthesis, namely AGPase and starch synthase, participate in the CHO metabolism pathway and are each encoded by four genes. During seed germination, more genes encoding AGPase were down-regulated than up-regulated. Among the four genes encoding susy in the sucrose decomposition pathway, more were up-regulated than down-regulated. In the amino acid metabolism pathway, the number of up-regulated genes encoding alanine aminotransferase increased as seed germination progressed, while the number of down-regulated genes encoding asparagine synthetase decreased during this time. Gene names, numbers and expression patterns in other pathways are listed in Fig. 5.

To further validate our RNA-Seq results, a total of 12 genes were selected for Real-time PCR. The fold-change values for these genes expression patterns indicated by histogram are presented in Additional file 1: Fig. S1. Line graph represents the fold change of RNA-Seq data. The RNA-Seq data and Real-time PCR results were highly consistent, which confirms the reliability of the transcriptome data.

### Changes in contents of some primary metabolism-related metabolites

**Fig. 6:**
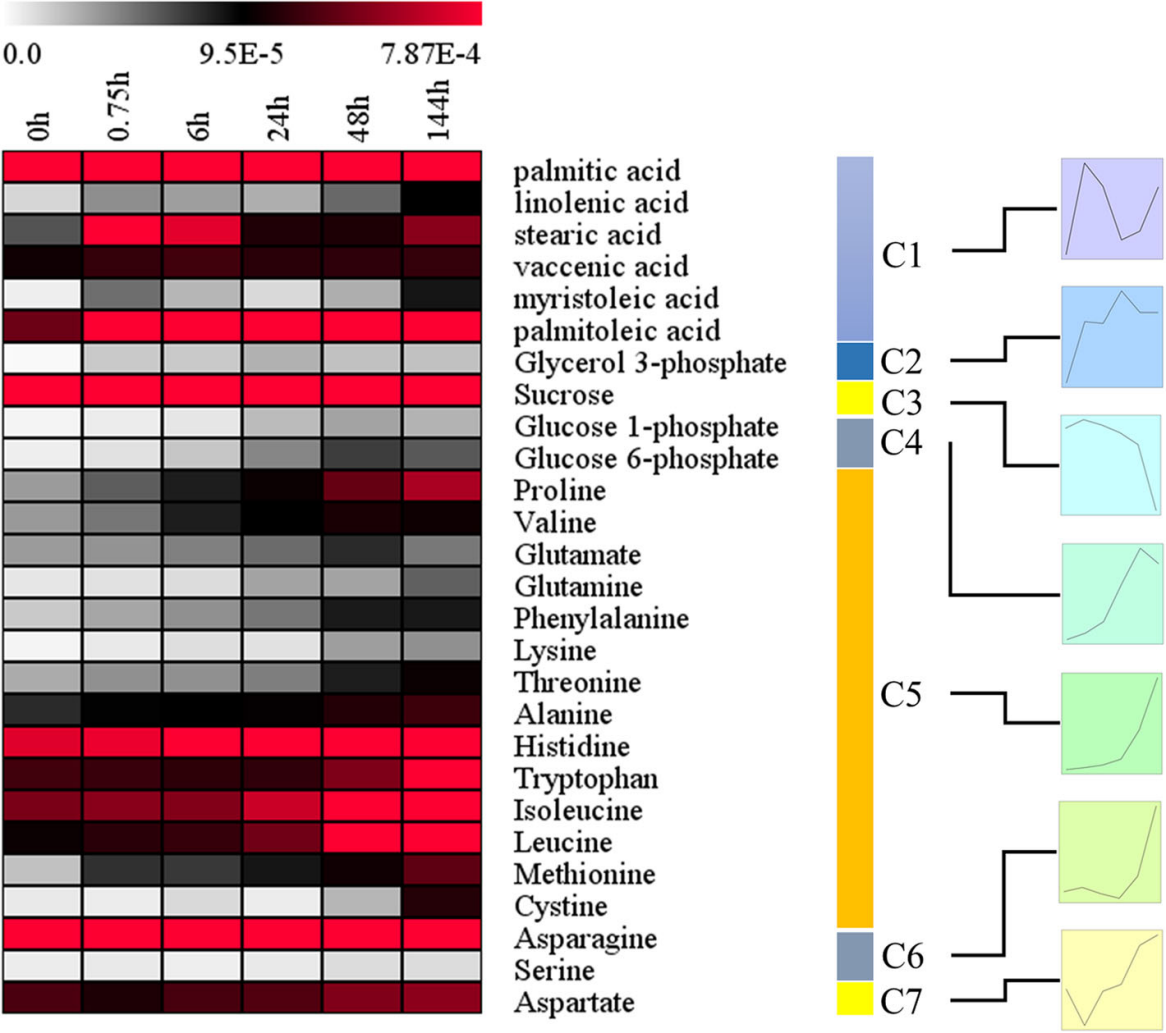
Changes in the contents of metabolites, such as lipids, sugars and amino acids. Left, heat map of changes in metabolite abundance during poplar seed germination; right, categorization of metabolites according to their patterns of change in abundance

To understand changes in primary metabolite levels during poplar seed germination, we detected amino acids, lipids and carbohydrates during different stages of seed germination using a metabolomics approach and classified them according to their content change patterns (Fig. 6). Lipid content increased during two stages, between 0.75 and 6 h and then after 144 h, while the content of glycerol 3-phosphate was elevated throughout germination. Sucrose, glucose 1-phosphate and glucose 6-phosphate are all carbohydrate metabolites. Among them, the content of sucrose, a storage substance, was high during seed germination and then decreased at 144 h. Glucose 1-phosphate and glucose 6-phosphate are the main components of phosphate hexose pools, and their contents significantly increased after 24 h. Most detected amino acids had increased contents during the late stage of seed germination; the exception was aspartic acid, which was present during the dry seed period and least abundant during the period of rapid water absorption.

### Changes in physiological index

**Fig. 7:**
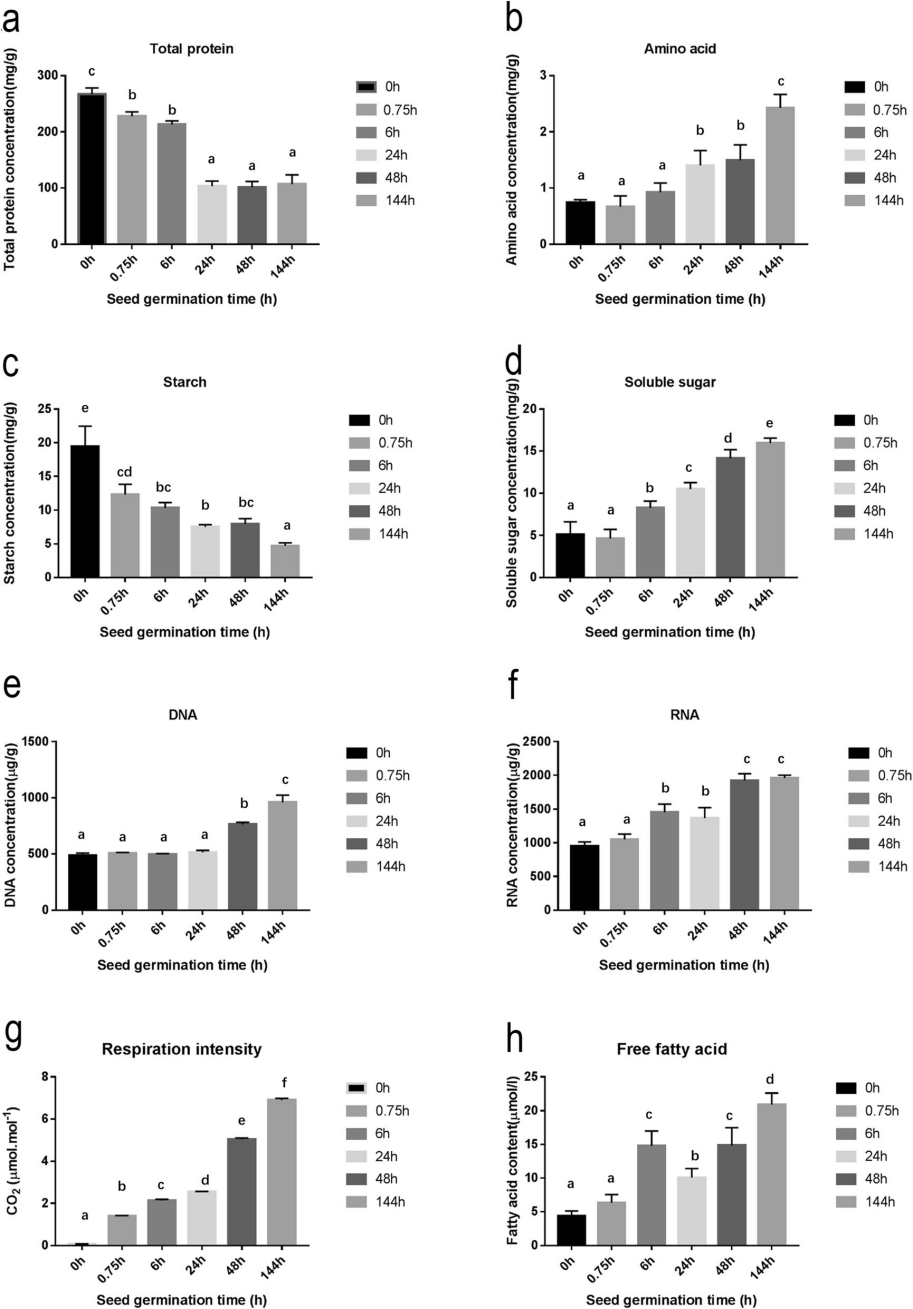
Analysis of changes in physiological parameters related to primary metabolism. (**a**–**h**) Changes in total protein content (**a**), free amino acid content (**b**), starch content (**c**) soluble sugar content (**d**), DNA content (e), RNA content (**f**), respiratory rate (**g**) and free fatty acid content (**h**) during poplar seed germination. In each plot, bars labelled with different lowercase letters are significantly different

Total protein and free amino acid contents at different stages of poplar seed germination are shown in Fig. 7a and b. As shown in Fig. 7a, the total protein content of seeds was significantly decreased at 0.75 h. At 24 h, the total protein content again decreased to approximately 103 mg/g and then remained relatively stable until approximately 144 h. The free amino acid content generally rose during seed germination; it was slightly decreased at 0.75 h and then increased significantly at 24 h, reaching approximately 1.4 mg/g. The decrease at 0.75 h may be related to cell membrane leakage, while the change in content around 24 h may be associated with the decomposition of storage proteins.

We also monitored changes in starch and soluble sugar contents during poplar seed germination. Our results are shown in Fig. 7c and d. Starch content decreased from 0 to 24 h, from approximately 19 to 7.54 mg/g; it then experienced a slight rise and subsequently dropped at 144 h. This pattern suggests that carbohydrate metabolism was relatively active during seed germination.

DNA and RNA are important macromolecules in living organisms. Changes in the contents of these two nucleic acids during seed germination are shown in Fig. 7e and f. In the early stage of seed germination, DNA content was relatively stable, remaining at 500 μg/g until 24 h and then increasing significantly at 48 h. RNA content began to increase at approximately 6 h, with a significant increase at approximately 48 h. The significant increase in RNA content prior to that of DNA may be the result of cell elongation preceding cell division during poplar seed germination.

Changes in respiratory intensity during poplar seed germination are shown in Fig. 7g. Respiratory intensity increased continuously during all the seed germination and post germination stages, which indicates that ATP synthesis began to gradually recover during early seed germination.

As shown in Fig. 7h, free fatty acid content first increased at 6 h, reaching 14.81 μmol/g, and then dropped to 10.08 μmol/g at 24 h. Fatty acid content again increased significantly at 48 h, reaching 14.84 μmol/g, which was basically consistent with the above-mentioned lipid metabolite trend.

## Discussion

Knowledge of transcription-level changes during poplar seed germination can aid understanding of the molecular mechanisms underlying seed germination in woody plants. Asexual reproduction in poplar has been widely studied, and the experimental techniques are quite reliable [18, 19]. In contrast, few reports have appeared on molecular mechanisms of poplar seed germination [15, 20–22]. We previously studied specific genes at different stages of poplar seed germination by weighted gene co-expression network analysis (WGCNA) and other methods and identified some candidate genes possibly playing important roles at certain germination stages. We were unable to determine, however, how transcription levels changed during poplar seed germination. In the present study, we resolved this question by another method, hypergeometric testing, and thereby laid a foundation for clarifying the molecular mechanisms responsible for poplar seed germination.

Previous investigations have shown that a series of complex molecular-level changes are involved in seed germination [9, 23]. In the present study, our analysis of transcripts from each germination stage allowed us to identify stage-specific genes involved in CHO, lipid, amino acid, nucleic acid and secondary metabolism. GO enrichment analysis, however, uncovered no significant enrichment of biological processes in any period (data no shown, AGRIGO, http://systemsbiology.cau.edu.cn/agriGOv2/) [24]. Even though we uncovered many genes specifically expressed during a given seed germination stage in this study, we speculate that the reason no enrichment was observed was because the number of genes participating in any given pathway was small.

During poplar seed germination, gene transcript levels were significantly enriched after 6 h, but no biological pathway enrichment was observed at 0.75 h. Previous studies have shown that relatively few significantly differentially expressed genes are present at 0.75 h compared with other germination stages [20]. Considering our other results, we believe that significant transcription-level changes occur at 0.75 h during poplar seed germination even though a systematic change in certain biological processes has not yet taken place.

An examination of the seed germination process in other species can provide significant information. Previous studies have shown that lipids play an important role in early germination. For example, lipid degradation-related genes begin to up-regulate their expression approximately 1 h after germination in *Hordeum vulgare* and *Oryza sativa* [25, 26]. In *Arabidopsis thaliana*, lipase and G3P shunt pathway transcripts increase during germination, while exogenous sucrose is unable to rescue mutants lacking triglyceride fatty acid enzymes and G3P shunt enzymes, thereby delaying and reducing germination [9, 27].

In our study, lipid metabolism transcriptional activity was elevated from 6 through 48 h (Fig. 4). In regards to metabolites, the relative abundance of fatty acids was high at 0.75 and 6 h and low between 24 and 48 h. Glycerol 3-phosphate had the highest relative abundance at approximately 24 to 48 h (Fig. 6). Total free fatty acid content peaked at 6 h, decreased at approximately 24 h and increased again at 48 h (Fig. 7). We believe that genes related to lipid metabolism are actively expressed between 6 and 48 h after seed germination. Hypocotyl elongation occurs at 24 h during seed germination; the decrease in free fatty acid content at this time may be related to the seed’s increasing requirements for these substances.

Although most seeds begin to mobilize starch reserves only after germination is completed, they begin to prepare for the mobilization and degradation of reserves during the first hour of germination. Amylase-related genes are up-regulated in *H. vulgare* and *O. sativa* at this stage [25, 26], and amylase activity is increased in germinating seeds and in vitro cotyledons in *Pisum sativum* [28]. In our study, CHO metabolism transcriptional activity was high during seed germination but decreased after 48 h. Glucose 1-phosphate and glucose 6-phosphate contents increased after 24 h, soluble sugar content increased significantly after 6 h, and starch content decreased significantly at 0.75 h. We thus infer that genes related to CHO metabolism play an important role in the early stages of seed germination.

Sugars inhibit the mobilization and use of amino acids during seed germination [29, 30]. Storage protein and amino acid utilization has been found to be enhanced under sugar depletion conditions during the latter stage of germination [31]. As can be seen in Fig. 4, the activity of protein metabolism-related genes reached its highest level at 6 h but decreased after 48 h. The activity of amino acid metabolism-related genes increased gradually during seed germination and was highest at 48 h. In regards to physiological levels, total protein content decreased and free amino acid content increased as the germination time was prolonged. As shown in Fig. 6, the contents of 15 of 17 amino acids detected by metabolomics increased at the late stage of poplar germination (all except aspartate and serine). We believe that changes in transcriptional levels of protein metabolism genes play a role in the middle and late stages of seed germination. The small amount of aspartate in dry seeds may be related to its storage function, while the increase in serine at 0.75 h may be due to the activity of glycerol 3-phosphate and glycolysis pathways.

ATP production in plants is accompanied by the release of carbon dioxide [32]. At the early stage of seed germination, mitochondrial function starts to recover, and transcripts related to energy generation and enzyme activities begin to increase. In this study, ATP synthesis-related genes were active at approximately 6 h, with their activities increasing again after 48 h. At the physiological level, the respiratory rate was high at 0.75 h, and another peak appeared at 48 h; these results indicate that the ATP synthesis function had recovered after 0.75 h of seed germination, with only partial gene activity required for recovery [33].

In general, the mobilization of primary metabolism-related genes likely proceeds in an ordered fashion. We hypothesize that the transcription of CHO metabolism-related genes is first activated to promote the decomposition of carbohydrates such as starch. Then, 6 h after germination, genes related to lipid metabolism are expressed, with those associated with protein metabolism and amino acid metabolism subsequently activated at the same time and transcribed until approximately 48 h. The results of our physiological and metabolic level analyses also support these inferences to some extent.

## Conclusions

In this study, we generated a timetable of transcription-level changes during the germination of seeds of *Populus × xiaohei*. Using this timetable, we were able to identify biological events occurring at the transcriptional level during different germination stages; we also compared the results of two differentially expressed-gene enrichment analysis methods, namely, fixed reference and continuous comparison systems, and found little difference between them. Primary metabolic processes, such as protein, amino acid, CHO and lipid metabolism, play important roles in seed germination. Our comparison of Σ*Z* values revealed that a sequence of changes in biological processes related to primary metabolism occur at the transcriptional level during poplar seed germination. Genes related to CHO metabolism are the first to be activated, followed by genes involved in lipid metabolism and finally those associated with protein metabolism. This order was further confirmed by metabolomics methods and the use of physiological indexes. In future research, we plan to further explore possible regulatory patterns at the transcriptional level.

## Methods

### Experimental conditions, data collection and analysis

Seeds produced in the same year from superior poplar trees (*Populus × xiaohei* T. S. Hwang et Liang) were selected from the greenhouse of Northeast Forestry University (Harbin, Heilongjiang, China), located at 45°72′N and 126°64′E. The plant materials used in this study are identified and preserved by the State Key Laboratory of Tree Genetics and Breeding (Northeast Forestry University), and the voucher specimen materials are also deposited in the State Key Laboratory of Tree Genetics and Breeding (Northeast Forestry University). The freshly harvested seeds were placed in a petri dish with filter paper and cultured at a constant temperature of 24 °C in dark. The sample period selection is described with reference to the previous [20], in short, the seed germination is divided into three periods according to the difference in fresh weight after water absorption, periods of rapid and slow water absorption were defined as stages 2 and 3, respectively, while the hypocotyl extension period was defined as stage 4, We collected samples at a total of six stages: stage 1 (0 h), stages 2 to 4 (0.75 h, 6 h and 24 h), and post-germination stages of cotyledon unfolding (stage 5, 48 h) and true-leaf unfolding (stage 6, 144 h). In the collection of samples, seeds were blotted with absorbent paper to remove surface moisture, quickly wrapped in tin foil and stored in liquid nitrogen and then kept in − 80 °C refrigerator.

RNA-seq and metabolome raw data were previously obtained at the State Key Laboratory of Tree Genetics and Breeding (Northeast Forestry University, Harbin, China), which were obtained using the RNA-seq technique on the Illumina HiSeq 2500 platform (Illumina) with seed of poplar at different germination stages with three replicates for each stage, was used to study changes in transcription levels at different germination periods. The transcriptome data were re-annotated through an integrated analysis based on the annotation of the JGR database (version poplar 3.0) (https://phytozome.jgi.doe.gov/pz/portal.html). Gene expression levels were normalized using the FPKM method. Differentially expressed genes were identified in NOISeq using fold change ≥2 and *p* ≥ 0.8 thresholds [34]. The Mapman tool was used to assign transcript pathway categories and to annotate genes and metabolites [35]. The PageMan tool was used to identify categories significantly enriched in differentially expressed genes based on two-tailed hypergeometric testing.

Metabolome raw data with significant differences were identified using screening thresholds of VIP > 1 and *p* > 0.05 (t-test) [36]. Metabolites with significantly different abundances during at least one germination period were mapped using the Mapman tool [35], which resulted in the identification of metabolites related to lipid metabolism, CHO synthesis and degradation, and amino acid metabolism.

### Enrichment analysis

To reveal whether a biological pathway was active, all significantly differentially expressed genes were classified as up- or down-regulated, and the degree of enrichment of each biological pathway at a certain stage was represented with a *Z*-value. More specifically, the effect of up- and down-regulated genes on the activity of a given pathway was expressed as a relative *Z*-value (Σ*Z*) according to the equation Σ*Z* = *Z*_up-regulated gene_ - *Z*_down-regulated gene_, where *Z*_up-regulated gene_ and *Z*_down-regulated gene_ respectively represent the degree of enrichment of up- and down-regulated genes in the pathway. If Σ*Z* was greater than 0, the pathway was considered to be active, with a value greater than 1.96 indicating that the pathway was significantly active; similarly, a pathway with a negative value was inactive.

### RNA extraction and quantitative real-time PCR detection

Total RNA was extracted from approximately 100 mg of samples using pBIOZOL Total RNA extraction reagent (BioFlux, Tokyo, Japan) according to the manufacturer’s instructions. Extracted RNA (1 μg) was treated with RNase-free DNase I and then used for single-strand cDNA synthesis with a reverse transcription kit (SYBR Premix Ex Taq; Takara). Real-time PCR was carried out according to a SYBR Green fluorescence-based procedure using UltraSYBR Mixture reagents (CWBIO, Beijing, China). The PCR cycling protocol consisted of an initial denaturation at 95 °C for 10 min, followed by 45 cycles of 95 °C for 15 s and 60 °C for 1 min. After the final cycle, a melting curve analysis was performed over a temperature range of 55–95 °C in increments of 1 °C to verify the reaction specificity. Using the actin gene [37] as a constitutive reference, relative expression was measured by the 2 − ^ΔΔ^Ct method [38]. The primers used in this study are given in Additional file 1: Table S4.

### Physiological index determination

Soluble protein contents were analyzed using the Bradford method [39], and free amino acid contents were determined according to the method of Rosen [40]. Total RNA was isolated using Trizol reagent (Invitrogen, Carlsbad, CA, USA) and then incubated with 10 U DNase I (Takara, Dalian, China) for 30 min at 37 °C to remove genomic DNA. Genomic DNA was isolated from seeds using a plant DNA extraction kit (DP305; Tiangen, Beijing, China). The DNA and RNA were quantified using a Nanodrop 2000c spectrophotometer (Thermo Scientific, Waltham, MA, USA). Concentrations of soluble sugars and starch in roots and leaves were determined by the anthrone method of Yemm and Willis (1954) [41] with minor modifications [42]. Transpiration rates were measured on a LI-6400 system (LI-COR, Lincoln, NE, USA), according to the manufacturer’s instructions. Total free fatty acid contents of seeds were determined using a free fatty acid extraction kit (FFA-2-W, Comin, Suzhou, Jiangsu, China).

### Statistical analyses

All physiological index data were subjected to one-way analysis of variance and tested for significant differences between treatments using SPSS v20.0 (SPSS Inc., Chicago, IL, USA). Treatment effects were evaluated by Duncan’s test (*p* < 0.05).

## Supplementary information

**Additional file 1: Table S1.** Genes specifically expressed during seed germination and their annotations. **Fig. S1.** The RT-PCR validation of 12 differentially expressed genes during seed germination stages. The columns represent the RT-PCR relative expression pattern, data are presented as the mean ± SD (*n* = 3). The lines represent the RNA-Seq data results. **Table S4.** The primer sequences of RT-PCR.

**Additional file 2: Table S2.** *Z*-values of genes related to different biological processes based on two different comparison methods.

**Additional file 3: Table S3.** Relative *Z*-value data at each seed germination stage.

## Data Availability

All datasets generated or analyzed during this study are available from the corresponding author on reasonable request.

## Abbreviations

AGPase: ADP-glucose pyrophosphorylase
ATP: Adenosine triphosphate
CHO: Carbohydrate
FPKM: Fragments Per Kilobase of transcript per Million fragments mapped
G3P: Glycerol 3-phosphate
RNA-seq: RNA Sequencing
VIP: Variable importance in the projection
WGCNA: Weighted gene co-expression network analysis
