# Supplementary figures and images for: The transcriptional events and their relationship to physiological changes during poplar seed germination and post-germination
Source: BMC Genomics. 2019 Nov 4;20:801. doi: 10.1186/s12864-019-6180-5 (PMC6829952; doi:10.1186/s12864-019-6180-5)

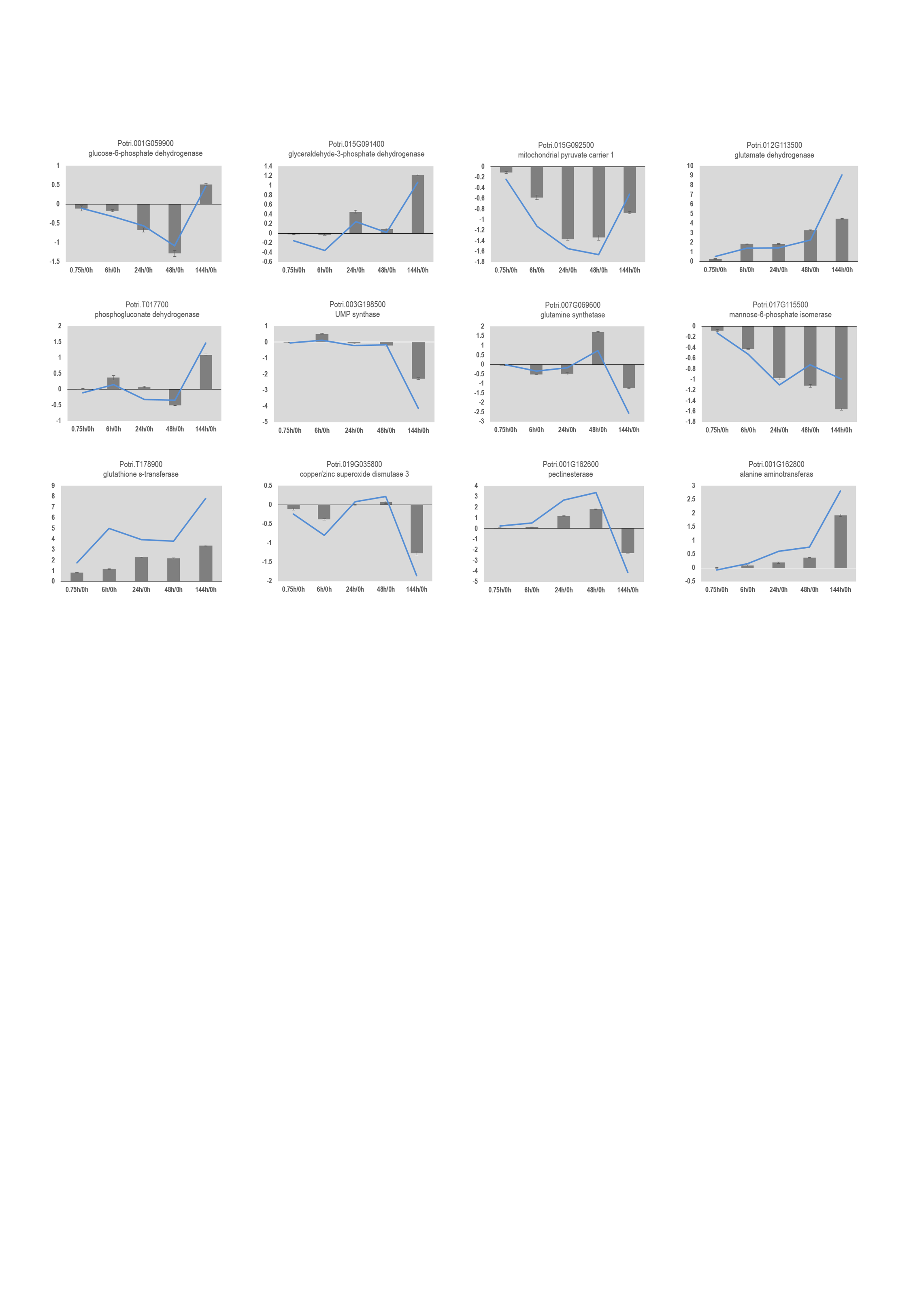

Supplement: Supplementary file 1 — Additional file 1: Table S1. Genes specifically expressed during seed germination and their annotations. Fig. S1. The RT-PCR validation of 12 differentially expressed genes during seed germination stages. The columns represent the RT-PCR relative expression pattern, data are presented as the mean ± SD (n = 3). The lines represent the RNA-Seq data results. Table S4. The primer sequences of RT-PCR. [file 12864_2019_6180_MOESM1_ESM.zip › Fig S1.tif]
